# Metabolomic Heterogeneity of Pulmonary Arterial Hypertension

**DOI:** 10.1371/journal.pone.0088727

**Published:** 2014-02-12

**Authors:** Yidan Zhao, Jenny Peng, Catherine Lu, Michael Hsin, Marco Mura, Licun Wu, Lei Chu, Ricardo Zamel, Tiago Machuca, Thomas Waddell, Mingyao Liu, Shaf Keshavjee, John Granton, Marc de Perrot

**Affiliations:** Latner Thoracic Surgery Research Laboratories and Division of Thoracic Surgery, Toronto General Hospital, University Health Network, University of Toronto, Toronto, Ontario, Canada; University of Louisville, United States of America

## Abstract

Although multiple gene and protein expression have been extensively profiled in human pulmonary arterial hypertension (PAH), the mechanism for the development and progression of pulmonary hypertension remains elusive. Analysis of the global metabolomic heterogeneity within the pulmonary vascular system leads to a better understanding of disease progression. Using a combination of high-throughput liquid-and-gas-chromatography-based mass spectrometry, we showed unbiased metabolomic profiles of disrupted glycolysis, increased TCA cycle, and fatty acid metabolites with altered oxidation pathways in the human PAH lung. The results suggest that PAH has specific metabolic pathways contributing to increased ATP synthesis for the vascular remodeling process in severe pulmonary hypertension. These identified metabolites may serve as potential biomarkers for the diagnosis of PAH. By profiling metabolomic alterations of the PAH lung, we reveal new pathogenic mechanisms of PAH, opening an avenue of exploration for therapeutics that target metabolic pathway alterations in the progression of PAH.

## Introduction

Pulmonary arterial hypertension (PAH) is a vascular disease characterized by persistent precapillary pulmonary hypertension (PH), leading to progressive right heart failure and premature death [1]. Pulmonary hypertension can either be idiopathic (sporadic-90%, familial-10%) or be the result of other conditions such as connective tissue disease, congenital heart disease, anorexigen use (dexfenfluramine), portal hypertension, and human immunodeficiency virus [1]. However, the pathological mechanisms underlying this condition remain elusive. Pulmonary artery endothelial cell (PAEC) dysfunction and structural remodeling of the pulmonary vessels are early features of PAH, characterized by a hyperproliferative and anti-apoptotic diathesis within the vascular wall of the resistant pulmonary arteries, leading to vascular lumen occlusion, right ventricular failure, and death. It has been reported that the PAH vascular remodeling process includes proliferation and migration of pulmonary artery SMCs, leading to medial hypertrophy and increased pulmonary vascular resistance [2], [3]. The local imbalance in vasoactive mediators as well as shear stress promotes proliferation and hypertrophy of endothelial and smooth muscle cells within pulmonary arterioles. Early stages of vascular remodeling include medial hypertrophy and hyperplasia, whereas the arterioles of patients with advanced PAH are characterized by complex plexiform lesions resulting from intimal hyperplasia [1], [4]–[6]. The terminal stage of PAH is characterized by a significant reduction in the cross sectional area of the pulmonary vasculature leading to right ventricular failure - a major factor for morbidity and mortality [4]. Recent evidence shows that abnormal metabolic pathways may also play a significant role in the development and progression of PAH [7]. A similar metabolic change has been identified as a feature of malignant tumor transformation displaying characteristics similar to hyperproliferative PAECs in PAH [8], [9]. Moreover, it has been shown that mitochondrial oxidative phosphorylation with glucose uptake and utilization occurs in the pulmonary artery endothelium of PAH patients [10], increasing the likelihood that metabolic alterations in PAECs may be representative of disease development. Increased hemoglobin levels have been found in the PAH sample group without a history of diabetes or any other obvious metabolic diseases, indicating the impairment of whole-body glucose homeostasis in PAH [11]–[13]. In animal models with chronic hypoxia induced PAH, vascular changes that are characteristic of the disease have been directly linked to an imbalance between glycolysis, glucose oxidation, and fatty acid oxidation [14]. In addition, *in* vitro PA endothelial cell culture with disruption of the BMPRII gene also showed significant metabolomic changes [7]. These data from *in vitro* and animal models suggest that molecular transcript and metabolic reprogramming might play an important role in the molecular pathogenesis of the early or developing stage of pulmonary hypertension. Here, we provide direct evidence that metabolic heterogeneity exists in the human lung with severe PAH. Our results show specific metabolic pathways and genetic profiles with disrupted glycolysis, increased TCA cycle and fatty acid metabolites with altered oxidation pathways in the later stage of the human PAH lung, which suggest that metabolic disruptions may underlie progression of severity for PAH. These identified metabolites may serve as potential biomarkers for the diagnosis of PAH. In addition, by profiling metabolomic alterations of the PAH lung, we reveal new pathogenic mechanisms of severe PAH, which may differ from the earlier stage of PAH, opening an avenue of exploration for therapeutics that target metabolic pathway alterations in the progression of PAH.

## Materials and Methods

### Patient population and Clinical Characteristics

Global biochemical profiles were determined in human lung tissue and compared across 8 normal (47 +/− 15 years of age, 4 females) and 8 pulmonary arterial hypertension patients of PAH (5 idiopathic pulmonary hypertension (IPAH), 2 systemic lupus (SLE)-PAH, 1 congenital heart disease (CHD)-PAH and Eisenmenger's syndrome-PAH;40 +/− 12 years of age, 5 females). All patients provided written informed consent, in accordance with the Declaration of Helsinki, for research protocols approved by the University Health Network (UHN) Research Board (#110932_AE). Eligibility criteria included end stage PAH patients who went through lung transplantation. Lung samples were obtained from the recipient lung at the time of lung transplantation. Control lung samples were obtained from normal tissue of cancer patients undergoing surgery lobectomy. The lung samples were snap frozen in the operating room and stored in −80°C prior to sample analysis. The PAH patient cohort included patients who had the WHO group l classification of pulmonary hypertension according to the fifth World Symposium on Pulmonary Hypertension [1]. Pulmonary hypertension was diagnosed by right heart catheterization performed for clinical care (with mean pulmonary artery pressures of 56±9 mm Hg, systolic pressure of 88±16 mmHg, and diastolic pressure of 34±14 mmHg). All patients provided written informed consent, in accordance with the Declaration of Helsinki, for research protocols approved by the institutional review boards of the University Health Network (UHN).

### Metabolomic profiling

Metabolomic analysis was performed as previously described [7]. Briefly, samples were prepared using the automated MicroLab STAR® system (Hamilton Co, Reno, NV, USA). A recovery standard was added prior to the first step in the extraction process for quality control purposes. Samples were prepared using the aqueous methanol extraction process to remove the protein fraction while allowing maximum recovery of small molecules. *Metabolomic performance:* The resulting extract was divided into four fractions: one for analysis by UPLC/MS/MS (positive mode), one for UPLC/MS/MS (negative mode), one for GC/MS, and one for backup. Samples were placed briefly on a TurboVap® (Zymark) to remove the organic solvent. Each sample was frozen and dried under vacuum conditions. Samples were then prepared for the appropriate instrument, either UPLC/MS/MS or GC/MS.

### Ultrahigh performance liquid chromatography/Mass Spectroscopy (UPLC/MS/MS)

The LC/MS portion of the platform was based on a Waters ACQUITY ultra-performance liquid chromatography (UPLC) and a Thermo-Finnigan linear trap quadrupole (LTQ) mass spectrometer, which consisted of an electrospray ionization (ESI) source and linear ion-trap (LIT) mass analyzer. The sample extract was dried then reconstituted in acidic or basic LC-compatible solvents, each of which contained 8 or more injection standards at fixed concentrations to ensure injection and chromatographic consistency. One aliquot was analyzed using acidic positive ion optimized conditions and the other using basic negative ion optimized conditions in two independent injections using separate dedicated columns. Extracts reconstituted in acidic conditions were gradient eluted using water and methanol containing 0.1% formic acid, while the basic extracts, which also used water/methanol, contained 6.5 mM ammonium bicarbonate. The MS analysis alternated between MS and data-dependent MS2 scans using dynamic exclusion. Raw data files were archived and extracted as described below.

### Gas chromatography/Mass Spectroscopy (GC/MS)

The samples destined for GC/MS analysis were re-dried under vacuum desiccation for a minimum of 24 hours prior to being derivatized under dried nitrogen using bistrimethyl-silyl-triflouroacetamide (BSTFA). The GC column was 5% phenyl and the temperature ramp was from 40° to 300°C in a 16 minute period. Samples were analyzed on a Thermo-Finnigan Trace DSQ fast-scanning single-quadrupole mass spectrometer using electron impact ionization. The instrument was tuned and calibrated for mass resolution and mass accuracy on a daily basis. The information output from the raw data files was automatically extracted as discussed below.

### Quality Control (QC)

Additional samples were included with each day's analysis. These samples included extracts of a pool created from a small aliquot of the experimental samples and process blanks. QC samples were spaced evenly among the injections and all experimental samples were randomly distributed throughout the run. A selection of QC compounds was added to every sample for chromatographic alignment, including those being tested. These compounds were carefully chosen so as to not interfere with the measurement of the endogenous compounds.

### Data extraction and compound identification

Raw data was extracted, peak-identified, and QC was processed using Metabolon's hardware and software. These systems are built on a web-service platform utilizing Microsoft's NET technologies, which run on high-performance application servers and fiber-channel storage arrays in clusters to provide active failover and load-balancing. Compounds were identified by comparison to library entries of purified standards or recurrent unknown entities. More than 2400 commercially available purified standard compounds have been acquired and registered into LIMS for distribution to both the LC and GC platforms for determination of their analytical characteristics.

### Statistical Analysis

Missing values (if any) were assumed to be below the level of detection. However, biochemicals that were detected in all samples from one or more groups, but not in samples from other groups were assumed to be near the lower limit of detection in the groups in which they were not detected. In this case, the lowest detected level of these biochemicals was imputed for samples in which that biochemical was not detected. Following log transformation and imputation with minimum observed values for each compound, a Welch's two-sample t-test was used to identify biochemicals that differed significantly between experimental groups. Data analysis was based on statistical significance (as well as those approaching significance (0.05<p<0.10). Pathways were assigned for each metabolite in order to examine the impact of an increased or decreased metabolite on the overall pathway.

### Transcriptomic analysis

Global profiles were determined in human lung tissue and compared across normal and idiopathic pulmonary arterial hypertension patients. The total RNA lung tissue analyses were performed using Trizol extraction according to the manufacturer's instructions. Biotinylated cRNA were prepared according to the standard Affymetrix protocol from 6 ug total RNA (Expression Analysis). Following fragmentation, 10 ug of cRNA were hybridized for 16 hr at 45C on GeneChip Genome Array. GeneChips were scanned using the HuGene-1_0-st-v1 GeneArray Scanner G2500A. The data were analyzed with Partek Genomics Suite 6.6 using Affymetrix default analysis settings and global scaling as the normalization method. The value definition was set up using Partek Genomics Suite 6.6. Significantly changed genes were determined using a minimum difference in expression of at least 200 arbitrary Affymetrix units, and *P*<0.01 by t-test with a false discovery rate (FDR) of 2 fold. The database has been submitted to NCBI/GEO and has been approved and assigned a GEO accession number, GSE53408.

### Immunoblotting

Protein concentrations were determined using the BCA protein assay (Pierce, IL, USA). Equal amounts of the protein lysates were separated by SDS-PAGE and transferred onto nitrocellulose membranes. The membranes were incubated overnight at 4°C with the following antibodies from Abcam^R^: anti-G6PC3 (1∶1000); anti-Lactate-Dehydrogenase-B (1∶500); anti-ALDH18A1 (1∶500). After washing with TBS-Tween, the blots were incubated for 60 min at room temperature with horseradish peroxidase-conjugated antibodies, respectively: anti-rabbit antibody (1∶15,000; Sigma-Aldrich, St. Louis, MO). Signals from immunoreactive bands were visualized by fluorography using an ECL reagent (Pierce). The intensity of individual bands in the immunoblots was quantified using the NIH Image program.

### Immunohistochemistry

The sections of both PAH and normal lung tissue were fixed for 4 hours at room temperature with PBS made of 4% formaldehyde, permeabilized for 30 min in Triton X-100 (0.5% in PBS), and incubated with 5% nonfat skim milk in PBS for 90 min. Sections were incubated for 180 min at room temperature with antibodies for anti-G6PC3 (1∶1000); anti- Lactate-Dehydrogenase-B (1∶500); or anti- ALDH18A1 (1∶500). The sections were then incubated with biotinylated secondary antibody and visualized with DAB. Stained cells and sections were visualized with the Zeiss LSM 510 confocal microscope.

## Results

PAH lung samples displayed broad changes in glucose and fatty acid metabolism. Significant changes were also observed in the TCA cycle compared to control lungs. We also analyzed the microarray database and paid specific attention to enzyme related genes that control and regulate affected metabolic pathways.

### Profiling of gene array and metabolic analysis of the severe PAH lung showed a significant alteration of multiple interdependent metabolic pathways

**Figure 1:**
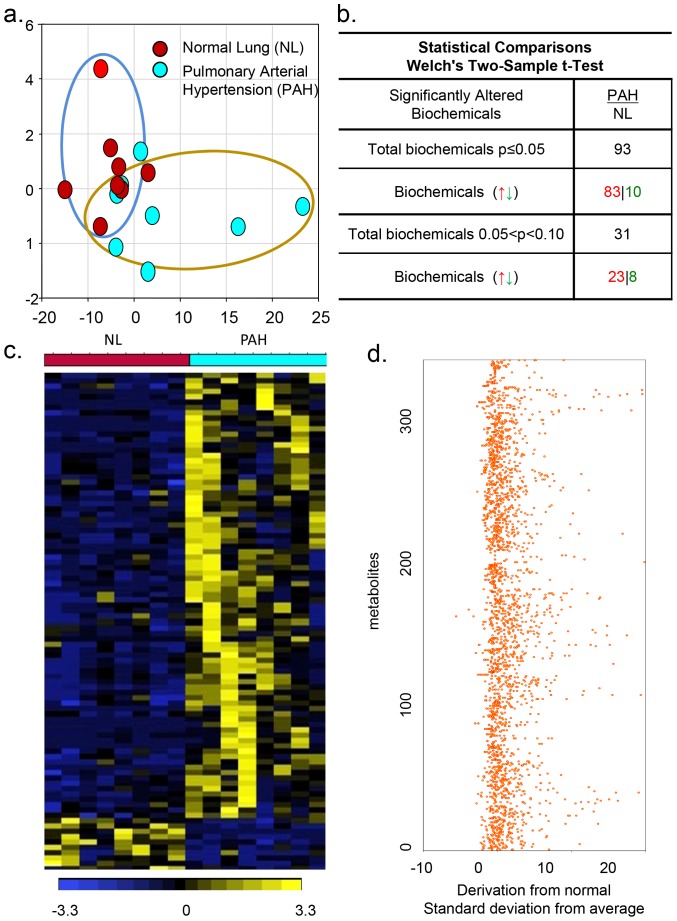
Metabolomic profiling of pulmonary hypertension. a), Principal component analysis (PCA) for metabolom PAH tissue exhibited a distinct metabolic signature in comparison to NL. b), Statistical comparisons using Welch's Two-Sample t-Test show significantly altered biochemicals in PAH samples (N = 8) compared with biochemical profiling in normal samples (N = 8). Interestingly, the biochemical profiles of PAH tissue exhibited higher levels of 93 altered metabolites compared with the normal lung (p≤0.05). c), Heat map that represents the non-supervised hierarchical clustering of 93 differential metabolites in PCA relative to normal sample data over eight PAH lungs (n = 8). Shades of light yellow/blue represent the increase and decrease of a metabolite, respectively, relative to the median metabolite levels. d) z-score plots show the 376 metabolites data that were normalized to the mean of the normal samples (truncated at 25).

PAH tissues exhibited a distinct metabolic signature in comparison to the normal lung (NL), as shown in the principal component analysis (PCA). Interestingly, the biochemical profiles of PAH tissue showed a separation compared to control patients (**Figure 1a**). In a simultaneous multiplexed mass spectrometric quantification of several hundred small molecule metabolites in the PAH lung, 376 small molecule metabolites were found in PAH lung samples compared to normal lung samples (**Figure 1b**). Among these molecules, ninety three biochemicals from the PAH lung were significantly upregulated (n = 83) or down-regulated (n = 10) compared with respective metabolites from the normal samples (*P*-value <0.05; FDR 1.1%). Thirty-one additional metabolites showed a trend towards up-regulation (n = 23) or down-regulation (n = 8). These multiple metabolic changes in PAH reflect an important metabolic distinction of pulmonary hypertension in the heat map that represents the none-supervised hierarchical clustering (**Figure 1c**).Z-score plots show the 376 metabolites data that were normalized to the mean of the normal samples (truncated at 25, **Figure1d**) [15]. Collectively, PAH tissues were marked by a unique pattern of global metabolomic heterogeneity compared to healthy subjects.

### Abnormal cellular glycolysis in the severe PAH lung

**Figure 2:**
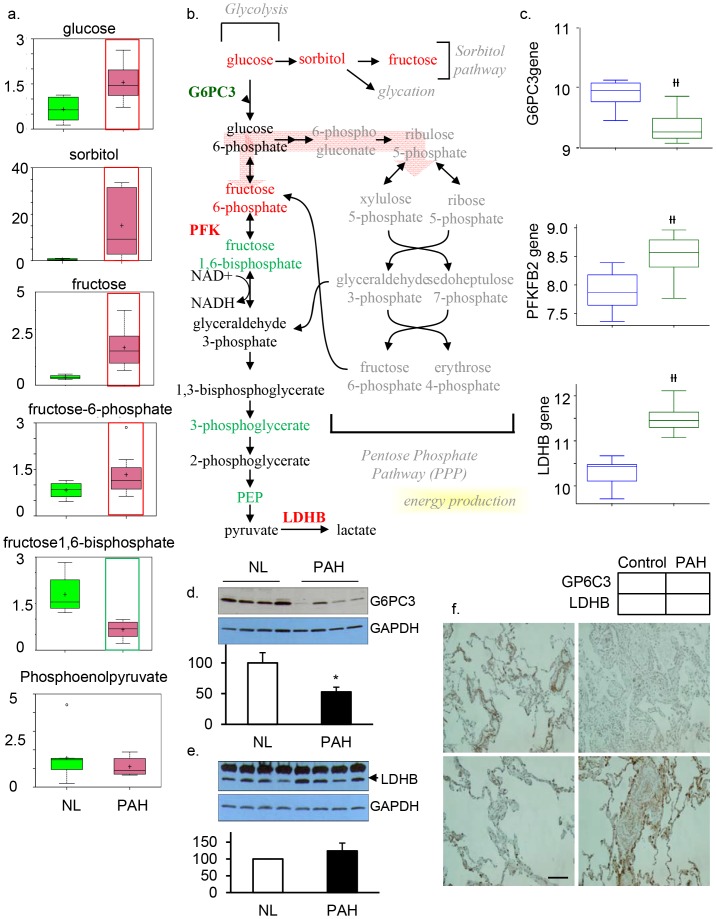
Glycolysis is significantly upregulated in the PAH lung. a) In all graphs, data for normal lung are shown in green boxes and data for the PAH lung are represented in pink boxes. Quantities are in arbitrary units specific to the internal standards for each quantified metabolite and normalized to protein concentration (N = 8 for each box). PAH patient samples exhibited higher levels of glucose, sorbitol, fructose, and fructose 6-phosphate. This metabolic disruption can contribute to the formation of advanced glycolytic end products that have been shown to directly contribute to the severity of PAH. b) The classical glycolysis/pentose/energy pathways are shown. c)Three genes encoding G6PC3, PFKFB2, and LDHB were significantly changed in PAH lung compared with NL, as shown in all graph) (G6PC3 (p = 8.54e−05), PFKFB2 (p = 2.6e-04) and LDHB (p = 2.19e−09). d) Western Blot analysis of G6PC3 (n = 4/each group) and e) LDHB expression in normal and PAH lungs (n = 4/each group). Lung lysate (20 ug per lane) was loaded and immunoblotted with antibody against G6PC3 or LDHB and GAPDH (loading control). Consistent with a significant decrease of G6PC3 and an increase of LDHB gene expression in PAH, protein expression for G6PC3 (39KD) was significantly decreased while LDH (37KD) was significantly increased in PAH lungs compared with NL lungs. Densitometric analysis of G6PC3 and LDHB were normalized to the intensity of the respective GAPDH band. Data are expressed as mean ±SD (n = 4). *P<0.05 versus NL. f), Representative images of G6PC3 positive immunostaining in the collagen fibers of pulmonary vascular tissue, which was decreased in PAH. Increased LDHB positive staining was found in pulmonary vascular smooth muscle tissue in the PAH lung (bar  =  1∶400).

Glucose metabolism plays an important role in the vascular remodeling process in PAH, since glucose is critical for the generation of cellular energy, nucleic acids, and biomass [13]. Therefore, we focused on glucose metabolites, gene encoding enzymes, and enzyme proteins that were progressively altered in glycolysis among PAH samples compared to the controls. PAH patients exhibited higher levels of glucose, sorbitol, fructose, and fructose-6-phosphate, suggesting the shuttling of glucose metabolism towards the sorbitol pathway (**Figure 2a**). Although higher levels of fructose 6-phosphate were observed in PAH samples, multiple late-stage glycolytic intermediates including fructose 1,6-bisphosphate, 3-phosphoglycerate, and phosphoenolpyruvate (PEP) were reduced in these tissues, indicating a disruption of glycolysis in PAH (**Figure 2b**). In conjunction with our metabolomics study, we also performed a molecular analysis. Gene microarray analysis showed that the gene encoding glucose 6-phosphatase subunit C3 (G6PC3), a key enzyme in the homeostatic regulation of blood glucose levels, was significantly decreased in the PAH lung (**Figure 2c**). G6P hydrolyzes glucose-6-phosphate and results in the creation of a phosphate group and a free glucose molecule. In agreement with findings from our metabolomic and microarray analyses, protein analysis showed that the expression of G6PC3 (38KD) was significantly decreased in PAH (**Figure 2d**). Immunohistochemistry showed that G6PC3 was found in collagen fibers around pulmonary vascular smooth muscle cells in the normal lung, and G6PC3 levels had decreased in collagen fibers of the PAH lung. Moreover, increased levels of fructose 6-phosphate in PAH lungs (**Figure 2f**) led us to believe that altered levels of fructose 6-phosphate may be indicative of a change in phosphofructokinase (PFK) activity. Indeed, our gene array analysis showed that PFK, specifically the 6-phosphofructo-2-kinase/fructose-2, 6-biphosphatase 2 (PFKFB2) gene, was significantly expressed in PAH compared to the normal control (**Figure 2c**). In addition, we found that the PAH lung had significantly increased gene expression for lactate dehydrogenase B (LDHB, **Figure 2c&e**), which catalyzes the interconversion of pyruvate to lactate with concomitant interconversion of NADH to NAD^+^ when oxygen is absent or in short supply. Increased levels of PFKFB2 and LDHB plus deceased G6PC3 at both genetic and protein levels may be the result of feedback mechanisms due to disrupted glycolysis and excessive intracellular and extracellular glucose levels. Together, these findings suggest that there is reprogramming of glucose metabolism in the severe PAH lung, leading to disrupted glucose uptake and altered glycolysis. Changes in glucose metabolism may contribute to the pathology of the disease by promoting vascular cell proliferation and vascular remodeling.

### Increase of 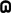-oxidation in dicarboxylic fatty acids and up-regulation of lipid oxidation in PAH

**Figure 3:**
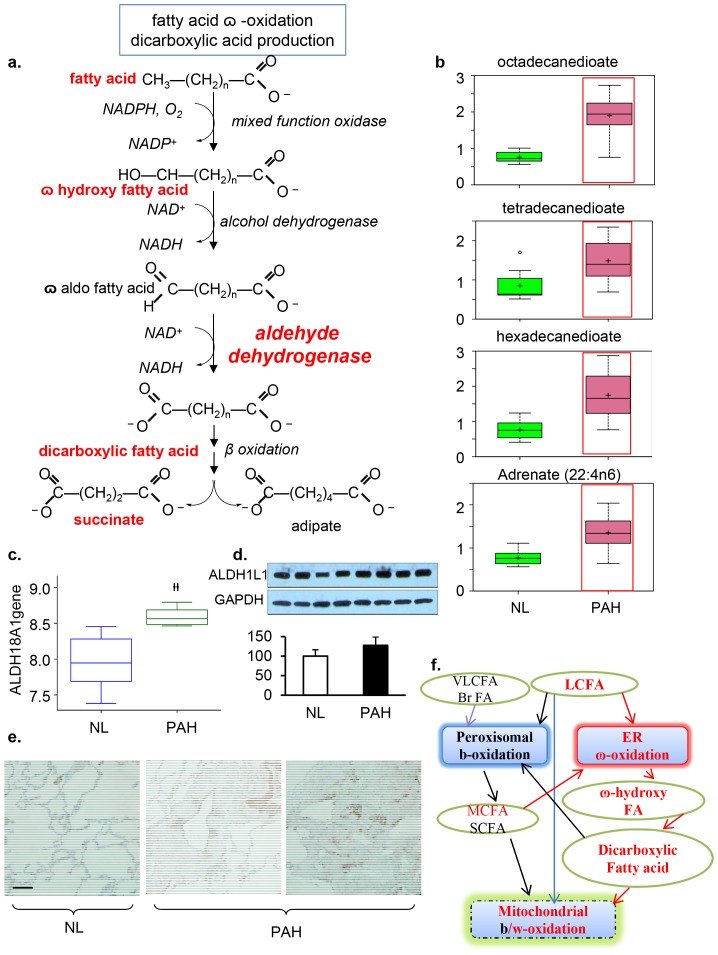
Mitochondrial β–Oxidation Disturbance. a), fatty acid 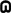
–oxidation for dicarboxylic acid production. b) In PAH tissue, there was a significant accumulation of the dicarboxylic fatty acids tetradecanedioate, hexadecanedioate, and octadecanedioate. An accumulation of adrenate (a pro-thrombotic lipid) can cause obstructions that may serve as a biomarker for the disorder. These changes in the lipid profile potentially reflect altered mitochondrial fatty acid oxidation. c), Gene array analysis showed that the gene encoding aldehyde dehydrogenase 18 family, member A1(ALDH18A1) was significantly increased in PAH (p = 1.87e4). d) Western blot analysis of ALDH1L1 expression in normal and PAH lungs. Lung lysate (20 ug per lane) was loaded and immunoblotted with antibody against ALDH1L1 and GAPDH (loading control). Protein expression of ALDH1L1 (40KD) was slightly increased in PAH lungs compared with NL lungs. Densitometric analysis of ALDH was normalized to the intensity of the respective GAPDH band. Data are expressed as mean± SD (n = 4). *P<0.05 versus NL. e) ALDH positive immunostaining in pulmonary vascular smooth muscle cells and endothelial cells in pulmonary vessel of the PAH lung. Representative micrographs of immunostaining of PAH lung sections with anti–ALDH in the pulmonary vascular endothelial cells (bar  =  1∶400). f) Dicarboxylic fatty acids are generated when the terminal methyl group of a fatty acid is converted into a carboxyl group and are often indicative of alterations in - **ω** oxidation. The catabolism of fatty acids typically occurs via β-oxidation in the peroxisomes and/or mitochondria. In contrast, fatty acid 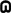-oxidation occurs in the smooth endoplasmic reticulum and often serves as a rescue pathway for pathological conditions such as PAH when β-oxidation is disrupted.

Dicarboxylic fatty acids are generated when the terminal methyl group of a fatty acid is converted into a carboxyl group (**Figure 3a**). The catabolism of fatty acids typically occurs via β-oxidation in the peroxisomes and/or mitochondria under normal conditions[16], [17]. Our metabolon data showed a significant accumulation of dicarboxylic fatty acids, in particular, tetradecanedioate, hexadecanedioate, and octadecanedioate in PAH tissue, suggesting that the fatty acid metabolic pathway had been altered to increase 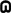-oxidation in the smooth endoplasmic reticulum in addition to β-oxidation in the peroxisome or mitochondria of the PAH lung (**Figure 3b**). To explore this finding further, we performed a gene array analysis and found that the gene encoding aldehyde dehydrogenase 18 family, member A1(ALDH18A1), a major enzyme in 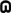-oxidation, was significantly over expressed in the PAH lung (p = 0.000187) (**Figure 3c**). Accordingly, protein expression of ALDH was also increased in the lung lysate (p = 0.04; **Figure 3d**). In addition, ALDH was highly expressed in human smooth muscle cells and endothelial cells (**Figure 3e**). Both metabolomical and genetic results indicate that 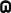-oxidation may serve as the major oxidation pathway for fatty acids when β-oxidation is no longer sufficient to supply ATP as a critical source of energy for the vascular remodeling process in PAH (**Figure 3f**).

**Figure 4:**
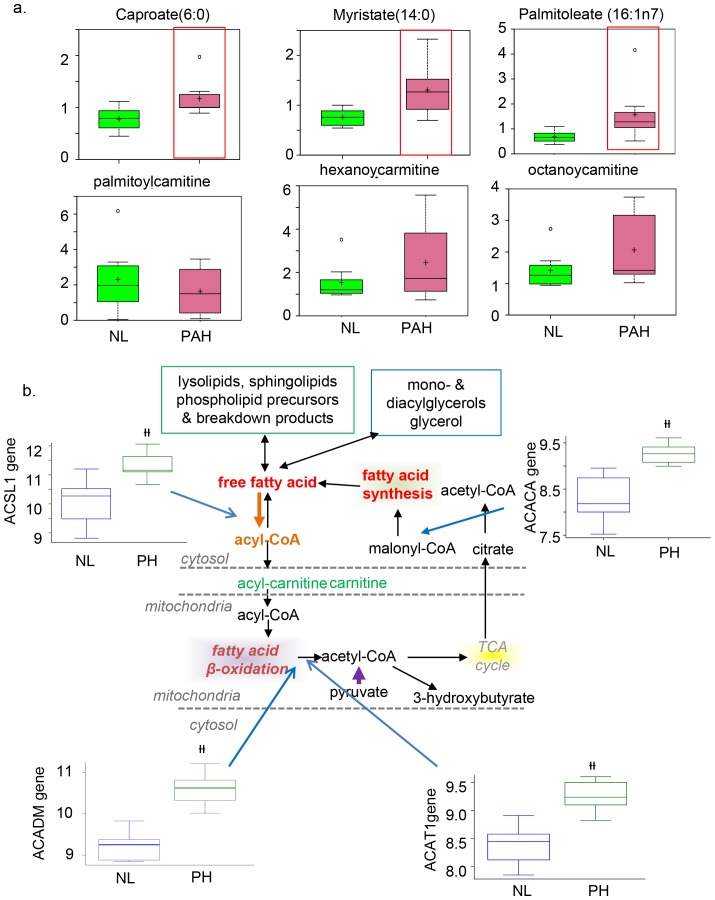
Carnitine metabolism and fatty acid oxidation are significantly increased in the PAH lung. Multiple carnitine metabolites that flow into the fatty acid oxidation pathway are shown. Gene array shows that expression of genes coding for the key enzymes that catalyze particular steps in the pathway are significant increased, specifically Fatty Acetyl CoA synthase L1 (ACSL1); Acyl-CoA dehydrogenases isoforms M (ACADM; Acetyl –Coa Acetyl transferase 1 (ACAT1); Acetyl CoA Carboxylase isoform A (ACACA) short chain, medium chain, short/branched chain, and very long chain; and malonyl CoA decarboxylase. Metabolic intermediates for which significant differences were detected between NL and PAH are shown with a red frame (p<0.05); N = 8 for each box).

In PAH tissue, there was also an accumulation of adrenate (a pro-thrombotic lipid that can cause obstructions). Long- and medium-chain free fatty acid products (including caproate, caprylate, myristate, and palmitoleate) accumulated in PAH tissues compared to control lung (**Figure 4a**). The increased lipid profile in PAH potentially reflects mitochondrial fatty acid oxidation. In correlation with the mebobolomics finding, we found that four genes that encode the enzymes fatty acetyl CoA L1 (ACSL1), Acyl-CoA dehydrogenases (ACADM), Acetyl –Coa Acetyl transferase1 (ACAT1), and Acetyl CoA Carboxylase (ACACA) were all significantly highly expressed (**Figure 4b**).

### Intermediate and enzyme encoded genes were significantly increased in the TCA cycle

**Figure 5:**
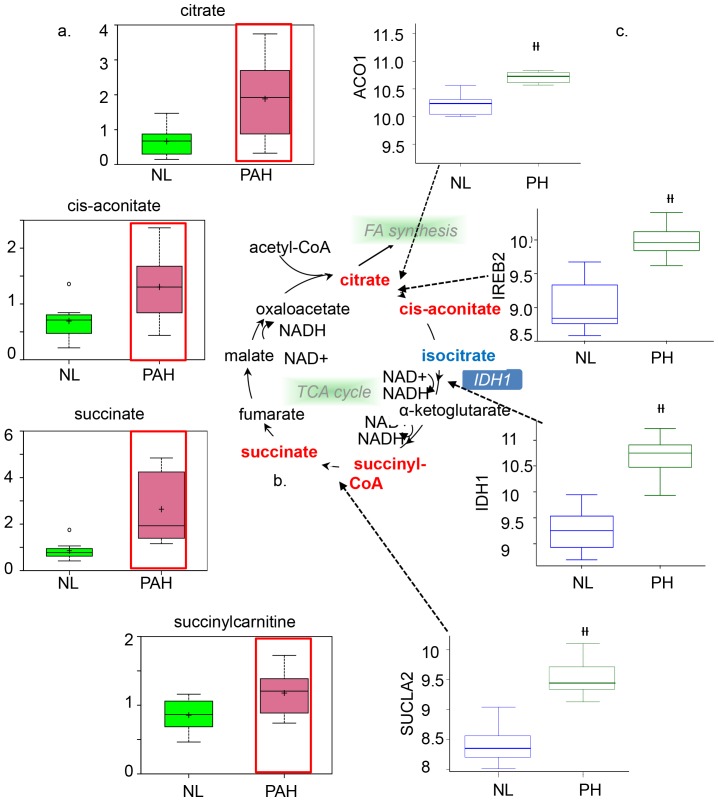
Upregulated TCA cycle in PAH lung samples. a) Data for metabolic intermediates in the normal lung are shown in green boxes and data for PAH are represented in pink boxes. PAH patients exhibited higher levels of citrate, cis-aconitate, succinate, and succinyl carnitine. b) The pathway for the tricarboxylic acid (TCA) cycle, the metabolic pathway for glucose oxidation in the mitochondria, is shown. Upregulated metabolic intermediates (citrate, cis-aconitase, succinyl-CoA, and succinate) are shown in red. c) Analysis of gene expression for key enzymes of the TCA cycle. Data from normal lung are represented in blue and data from PAH lung are shown in green. Aco1 (p = 1.068×10-3) andIREB-2were upregulated in the PAH lung sample (p = 1.59e-07), as well as IDH1 for converting isocitrate to α-ketoglutarate with the interconversion of NADP+ to NADPH (p = 4.96×10-9). SUCLA-2, which is one of two genes that encode succinate-CoA ligase for the conversion of succinyl-CoA to succinate with an interconversion of GDP to GTP, was upregulated in PAH patients compared to the normal control (p = 4.17×10-8).

In the TCA cycle, most intermediates were significantly increased in the PAH lung, including citrate and cis-aconitate (**Figure 5a**). Aconitase is the enzyme that catalyzes the formation of cis-aconitate from citrate. One of the two isoforms of aconitase is the iron−responsive element binding protein 1(Aco1) in the cytoplasm. Genetic analysis showed that Aco1 was more highly expressed in PAH (**Figure 5b**). The second isoform of aconitase, iron−responsive element binding protein 2 (IREB2), helps to control iron metabolism by binding to mRNA to repress translation or degradation. IREB-2 was also significantly increased in the PAH lung (**Figure 5b**), suggesting increased aconitase enzymatic activity may play a significant role in the conversion of citrate to isocitrate

Other TCA metabolites, including succinate and succinyl carnitine, were also elevated in PAH (**Figure 5a**). In correlation with increased metabolites, SUCLA2, the gene encoding succinate –CoA ligase, was significantly highly expressed (p = 1.59e-7, **Figure 5b**). In addition, the gene encoding fumarate hydratase (FH, data not shown) was also significantly highly expressed in the PAH lung. Our results show higher gene expression of isocitrate dehydrogenase1 (IDH1, p = 4.96e-9) in the PAH lung, suggesting that cytoplasmic IDH plays a significant role in cytoplasmic NADPH production. Together, these findings suggest that increased metabolites and related gene expression in the TCA cycle are altered in PAH patients and may potentially reflect abnormalities in mitochondrial function.

## Discussion

This study was conducted to identify differences in molecular and biochemical profiles of lung tissue harvested from normal lungs and lungs from patients with advanced PAH in an effort to better understand the metabolic changes that occur in the progression of early to severe PAH. Various pathological changes occurring in pulmonary arteries, specifically in the terminal small arteries, can contribute to the development and progression of PAH [18]. Understanding how changes in gene and protein expression of altered metabolic pathways contribute to the pathogenesis of PAH may lead to the development of new biomarkers and novel therapeutics that target these pathways. Our study of PAH metabolism primarily focused on pathways that, when altered, can lead to the aberrant production or consumption of essential biomolecules such as glucose, amino acids, nucleotides, and lipids in severe PAH. Most importantly, we have shown that there is disrupted glycolysis in the cytoplasm, altered glucose metabolism through the TCA pathway in the mitochondria, and altered fatty acid metabolism through 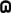-oxidation in the smooth endoplasmic reticulum (ER) in addition to β-oxidation in the mitochondria in the progression of severe PAH.

Interestingly, our results have shown that there is reduced glycolysis in the PAH lung compared to normal control, which is contrary to several previous studies [19]–[21], showing increased glycolysis as a significant characteristic of proliferating cells in PAH. The discrepancy between our findings to previous studies may be due to our usage of lung samples with severe PAH rather than lung samples with early or developing of PAH from previous works. Our results describe metabolic alterations that occur in the progression of PAH from the early to severe stage, where alterations in glucose metabolism through downregulation of glycolysis play an important role, while previous works likely focus on the metabolic alterations that occur in the initial onset or developing stage of PAH. Previous studies, based on hypoxia-induced PH in a relatively earlier/or developing stage of PH animal model (2–3 wks hypoxia) describes that the upregulation of hypoxia-induced factor (HIF-1α), in combination with HIF-1β, activates over 100 genes involved in metabolism [20]. In particular, there is increased glucose uptake via GLUT1 and GLUT3 as well as inhibition of the pyruvate dehydrogenase complex (PDC) by pyruvate dehydrogenase kinase (PDK) that normally oxidizes pyruvate to acetyl-CoA for the Krebs cycle [19], [21]. Other studies have shown that vascular endothelial proliferation in IPAH lesions displays pathological alterations that resemble characteristics of growing tumor cells in cancer [22]. These cells are characterized by the “Warburg effect”, as hyperproliferative tumor cells under hypoxic conditions use aerobic glycolysis with resultant changes in its mitochondrial redox state to escape apoptosis in the developing stage of the PH [9], [21], [23]. Results from previous studies that suggest for increased glycolysis had worked with experimental models of PH at the relatively early stage, such as *in vitro* studies using smooth muscle cells from animals exposed to 2–3 weeks of hypoxia or *in vitro* human pulmonary microvascular endothelial cells s(HMVEC-L) transfected with a BMPRII mutation [7]. In several of these studies, PH was induced by experimental measures and studies focused solely on one cell type, which would ignore possible cell-cell interactions that occur in the vascular remodeling process. In contrast to previous studies, our results were obtained from the severe human PAH lung rather than from animal models, which may be the underlying reason for the observation of reduced glycolysis. It remains elusive whether changes in metabolic pathways, for example, the rate of glycolysis, can reflect different stages in the progression of human pulmonary arterial hypertension. If so, such changes in glycolytic intermediates could serve as potential biomarkers for the diagnosis and prognosis of the disease. Our results suggest that there is a switch of energy usage with an overall decrease of glucose metabolism characterized by down regulated glycolysis, as well as excessive intracellular and extracellular glucose levels in the severe PAH lung. In addition, although glucose metabolism appears to be disrupted, excess glucose accumulation as a result of reduced glycolysis leads to the production of sorbitol, and, consequently, the potential formation of glycation products that can generate free radicals and trigger tissue damage [24]. Lactate levels did not significantly change, suggesting that excess glucose is used instead by the sorbitol pathway or pentose phosphate pathway. Based on our metabolomics and microarray data, we tentatively suggest that the human lung with advanced PAH does not produce high levels of lactate that are typically a signature trait of the Warburg effect in the earlier developing stages of PAH. Further experimentation based on the radioactive targeted approach [14] on the human PAH lung will clarify this issue.

Our study suggests that the process of vascular remodeling in PAH involves alterations in glycolysis in multiple cells, limited not only to SMCs but also includes endothelial cells and other tissues such as collagen fibers around the peri-vascular tissue. Lung samples from PAH patients exhibited higher levels of glucose, sorbitol, and fructose. By gene array and immunostaining, we showed that genes in vascular smooth muscle cells encoding the key enzymes for glycolysis, such as LDH-B, were significantly increased, whereas genetic expression of other key enzymes in the glycolytic pathway, specifically glucose-6-phosphatase subunit C3 (G6PC3) was significantly downregulated. Glucose-6-phosphate (G6P), a key rate-limiting metabolite in normal glycolysis and a substrate for G6PC3, can enter many pathways, including gluconeogenesis to produce glucose [25], glycogenesis for storing glucose, anaerobic glycolysis [26] to convert to pyruvate, or entrance to the pentose phosphate pathway for generating ribose-5-phsophate (R5P) for the synthesis of nucleotides and erythrose-4-phosphate (E4P) for the biosynthesis of aromatic amino acids. In particular, the enzyme glucose-6-phosphatase plays a major role in the gluconeogenesis process of dephosphorylating glucose-6-phsophate to generate glucose [27]. Our studies showed that G6PC3 was down-regulated in PAH at both the transcriptional and translational level (**Fig 2c,d,f**), suggesting that decreased expression of G6PC3 may be due to a decrease of G6P as a result of glucose being shuttled towards the sorbitol fructose pathway.

Despite a decrease in glycolytic key intermediates and enzymes, PFKFB2, an enzyme responsible for irreversibly converting fructose-6-phosphate (F6P) to fructose-1,6-bisphosphate (1,6-FBP) in the committed step of glycolysis was increased, perhaps in response to increased F6P levels, yet there was a decrease in the product fructose 1,6-bisphosphate in PAH lungs. An increase in PFKFB2 may be a feedback mechanism of decreased fructose 1,6-bisphosphate in an attempt to restore normal glycolysis, although protein levels of PFKB2 did not display significant changes. Our results also showed that the gene encoding lactate dehydrogenase B (LDHB) was highly expressed in the PAH lung. Further studies will be conducted to determine the specific roles of PFKFB2 and LDHB, and whether its upregulation is significant in promoting glycolysis as a countering mechanism for attenuating PAH.

With the understanding that fatty acid signaling is important during cholesterol metabolism and that the alteration of glucose and fatty acid signaling is a key factor for vascular remodeling in the development of PAH, we investigated the level of intermediate metabolites and expression of genes and enzymes of fatty acid metabolism in PAH lungs. Our results implied increased fatty acid metabolism due to increased expression of genes for beta oxidation, such as Acyl-CoA dehydrogenases isoforms M (ACADM) and Acetyl–Coa Acetyl transferase1 (ACAT1), suggest that fatty acid metabolism may play an important role in human PAH by switching the fuel of existing mitochondrial oxidative metabolism from glucose to fatty acids. Increased vascular remodeling in PAH can be achieved by increased fatty acid metabolism as well as by increased 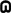-dicarboxylic fatty acid oxidation in the ER. Upregulation of omega oxidation, characterized by increased end products such as tetradecanedioate, hexadecanedioate, and octadecanedioate may compensate for the insufficient glucose metabolism. Fatty acid oxidation and glucose oxidation both produce mitochondrial acetyl-CoA. As a result, the rate of glucose oxidation has a direct and reciprocal effect on the rate of fatty acid oxidation and vice versa through the Randle cycle. The stimulation of fatty acid oxidation can replace glucose oxidation to produce high-energy cofactors at a more efficient rate. Therefore, our results suggest that vascular remodeling may rely primarily on fatty acid oxidation rather than on glycolysis, which is supported by an animal PAH model that showed attenuation of PAH upon inhibiting fatty acid oxidation due to a lack of malonyl-coenzyme A (MCD) expression [14]. Replacement of glucose oxidation with fatty acid oxidation also allows for increased production of ATP and NADPH in order to sustain rapidly dividing cells. Analyzing change in the level of intermediate metabolites and studying the regulation of specific enzymes in glycolysis, TCA, and fatty acid oxidation may provide a more accurate outline of the metabolic mechanisms in PAH. Ultimately, our result of increased fatty acid oxidation in PAH suggests that fatty acid inhibitors such as etomoxir and ranolazine trimetazidine may have beneficial effects in attenuating PAH.

The TCA cycle is the common pathway for the oxidation of carbohydrates, lipids, and selective amino acids. Our results concordantly showed that there is increased citrate and cis-aconitate at the beginning of the citric acid cycle, suggesting that there is an upregulation of the TCA cycle. As a result, metabolic intermediates of the TCA cycle are continually transported to the cytoplasm for increased fatty acid synthesis to produce energy for the vascular remodeling process. To support our speculation that metabolic changes in the TCA cycle contribute towards greater energy production, we also found increased conversion of succinyl-CoA to succinate, a process that normally produces high-energy GTP due to phosphorylation of GDP. In addition, the enzyme IDH1 is normally found in the cytoplasm and plays a key role in beta-oxidation of fatty acids in peroxisomes [27]. Increased genetic expression of IDH1 supports our results that there is increased beta-oxidation and that substrates for fatty acid oxidation are being shuttled towards omega-oxidation in the severe PAH lung. Our results also showed increased genetic expression of iron-responsive element binding protein (IREB-2), a cytoplasmic form of the enzyme aconitase that mediates the conversion of citrate to cis-aconitate. Our findings suggest that IREB-2 may be responsible for increased metabolic intermediates that were observed downstream of citrate in the TCA cycle.

Taken together, we explored the metabolome of PAH and characterized metabolomic signatures, which in the context of other molecular alterations may lead to a complete understanding of disease progression. Specifically, we identified that disrupted glycolysis in conjunction with increased fatty acid metabolism and an altered 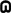-oxidation pathway directly regulates pathological vascular remodeling in the advanced stage of PH by means of transcriptional control of its regulatory enzymes. Fatty acid oxidation is a more efficient process compared to glycolysis for ATP production and would be the more ideal metabolic pathway for supplying energy for further vascular remodeling after plexiform lesions have developed. Identifying altered metabolites of glucose and fatty acid metabolism is ideal, as these metabolites may serve as potential biomarkers for diagnosing PAH, for making more accurate prognoses of the disease, and for monitoring PAH progression. Our results hold clinical significance for developing a combination of therapeutic techniques. With a better understanding of the metabolomic changes that occur during PAH, metabolic modulation therapy can be further developed to control vascular remodeling and cell proliferation for the treatment of PAH in its advanced stage. By reconsidering treatment strategies for PAH, we suggest that PAH can be attenuated by inhibiting glycolysis at the early stage of the disease and by inhibiting fatty acid oxidation towards the advanced stage of the disease. These metabolic interventions may open a new avenue of therapeutics that is less invasive for the treatment of PAH.

## Supporting Information

Figure S1 **Representative image of hematoxylin-eosin staining of lung from both control and PAH patients.** The control lung tissue from marginal zone of lung from tumor related lobectomy. Histological images of control shows that the control lung tissue is morphologically normal (bar ratio =  1∶100).
(TIF)
