# Supplementary figures and images for: Metabolomic Heterogeneity of Pulmonary Arterial Hypertension
Source: PLoS One. 2014 Feb 12;9(2):e88727. doi: 10.1371/journal.pone.0088727 (PMC3923046; doi:10.1371/journal.pone.0088727)

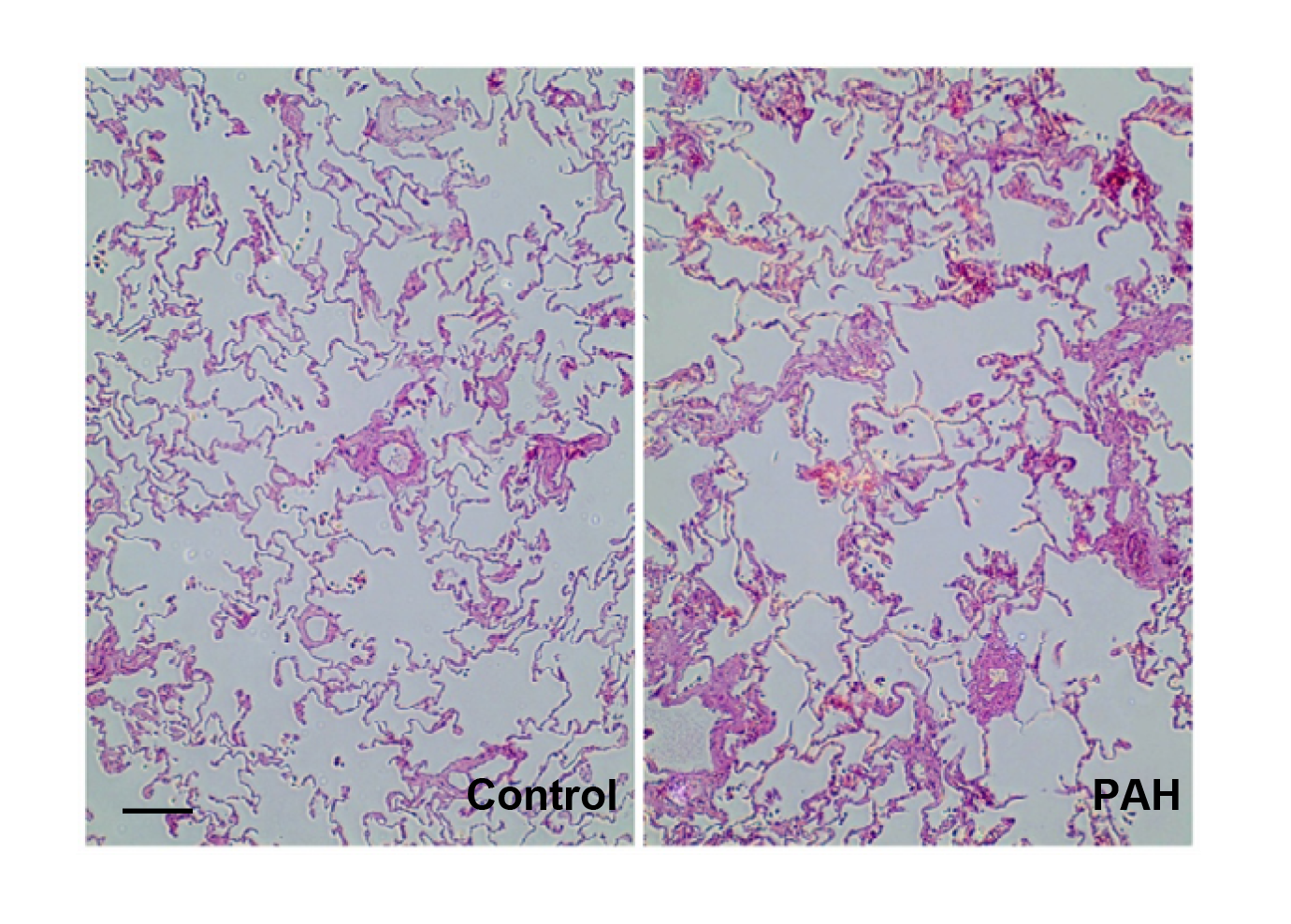

Supplement: Figure S1 — Representative image of hematoxylin-eosin staining of lung from both control and PAH patients. The control lung tissue from marginal zone of lung from tumor related lobectomy. Histological images of control shows that the control lung tissue is morphologically normal (bar ratio = 1∶100). (TIF) [file pone.0088727.s001.tif]
